# Mites inhabiting nests of wood warbler, *Phylloscopus sibilatrix* (Aves: Passeriformes), in the Wielkopolska National Park in western Poland

**DOI:** 10.1007/s10493-023-00792-5

**Published:** 2023-04-08

**Authors:** Alicja Laska, Ewa Puchalska, Martyna Mikołajczyk, Dariusz J. Gwiazdowicz, Andrzej Kaźmierski, Wojciech Niedbała, Jerzy Błoszyk, Ziemowit Olszanowski, Jakub Szymkowiak, Natalia Hałas, Lechosław Kuczyński, Anna Skoracka

**Affiliations:** 1 Population Ecology Lab, Faculty of Biology, Adam Mickiewicz University, Poznań, Poland; 2 Section of Applied Entomology, Department of Plant Protection, Warsaw University of Life Sciences – SGGW, Warsaw, Poland; 3 Department of Animal Morphology, Faculty of Biology, Adam Mickiewicz University, Poznań, Poland; 4 Department of Forest Entomology and Pathology, Poznań University of Life Sciences, Poznań, Poland; 5 Department of Animal Taxonomy and Ecology, Faculty of Biology, Adam Mickiewicz University, Poznań, Poland; 6 Department of General Zoology & Natural History Collections, Faculty of Biology, Adam Mickiewicz University, Poznań, Poland

**Keywords:** Wood warbler nests, Mesostigmata, Trombidiformes, Sarcoptiformes, Prevalence, Infestation

## Abstract

The wood warbler, *Phylloscopus sibilatrix* (Aves: Passeriformes), is a well-known model organism for studying bird migration, breeding habitat selection and nest predation. The nest acarofauna of this bird species has not been extensively studied so far. To provide a comprehensive report on mite species inhabiting wood warbler nests and to assess infestation parameters (prevalence, intensity, and abundance) for mite species and orders, we collected 45 nests of this bird species in the Wielkopolska National Park in western Poland. Analyses revealed a huge diversity (198 species) of mites inhabiting wood warbler nests. We found individuals belonging to the Mesostigmata, Trombidiformes and Sarcoptiformes. The Trombidiformes, represented in our study only by the Prostigmata, achieved statistically significantly lower intensity and abundance, compared to representatives of other orders. However, the number of recorded prostigmatid species was high (65). The most common were: *Stigmaeus sphagneti* (22 nests), *Stigmaeus longipilis* (16), *Eupodes voxencollinus* (15), *Cunaxa setirostris* (14), *Stigmaeus pilatus* (11), and *Linopodes* sp. 2 (10). The prevalence of Mesostigmata and Sarcoptiformes was equal, reaching 91.1%. Most of Gamasina (Mesostigmata) species found in this study were more characteristic of the soil environment and forest litter than bird nests, but there was also a typical bird parasite, viz. *Ornithonyssus sylviarum.* None of the observed species of Uropodina (Mesostigmata) or Oribatida (Sarcoptiformes) was typical for bird nests. Among the Uropodina, the highest parameters of nest infestation were achieved by *Oodinychus ovalis*, whereas among the Oribatida, they were achieved by *Metabelba pulverosa.* We discuss the importance of wood warbler nests for mite dispersal, survival and reproduction.

## Introduction

Mites are among the most diverse groups of invertebrate taxa and occupy a wide range of environments. Among many different microhabitats, mites inhabit nests and burrows of vertebrates where they act as ectoparasites, free-living predators, or as edaphic or coprophilous organisms (e.g., Proctor and Owens 2000; Mašán and Stanko 2005; Celebias et al. 2019). The mites common and typical for birds of nests and small mammals are called nidicolous (Napierała and Błoszyk 2013). To date, most attention has been paid to mite fauna inhabiting nests of mammals (e.g., rodents, moles) and birds, such as storks, eagles, owls, pigeons, sea birds, and some passerine species (Kaźmierski 1998a; Gwiazdowicz et al. 1999; Kristofik et al. 2002, 2005; Błoszyk et al. 2005; Pilskog et al. 2014; Napierała et al. 2016; Kaminskienė et al. 2017; Kaźmierski et al. 2018, 2021; Celebias et al. 2019). Those studies showed that the type of nest and the period when the nest is used play an important role in shaping mite assemblage structure. Mites are more abundant in yearly reused nests than in 1-year nests, as the latter are more ephemeral microhabitats, available only for a short time, i.e., during birds’ breeding period (e.g., Mašán et al. 2014).

Bird nests amaze us with a diversity of forms, occupied places, structures and building materials used, and provide various abiotic and biotic conditions for invertebrates. For example, the scrape-nest represents the simplest type of nest built in the ground as a shallow depression for the birds to lay their eggs. Birds that build scrape-nests tend to have precocial chicks that are able to leave the nest quickly after hatching (Campbell and Lack 1985; Reid et al. 2002). In nests of this type, the abundance of mites and the complexity of their communities are rather low (Ambros et al. 1992). In contrast, burrow nests and mound nests provide shelters that allow rapid mite development and reproduction, and in nests of these types, mite densities are usually high (Woodroffe 1953; Ambros et al. 1992; Fend’a 2010). Mound nests are often made from mud, branches, sticks, twigs, and leaves. When organic material of the nest begins to decay, the compost pile heats up and gives off heat to incubate the chicks (Campbell and Lack 1985; Hansell and Overhill 2000; Deeming and Reynolds 2015). Probably such conditions make mound nests attractive and suitable for invertebrates, including mites as has been shown for pigeon nests (Woodroffe 1953).

Mites inhabit also cup nests (Møller 1990; Mašán et al. 2014), platform nests (Gwiazdowicz et al. 1999), natural cavity nests (Pung et al. 2000), and artificial nest boxes (Stamp et al. 2002; Błoszyk et al. 2016) used by cavity nesters when natural cavities cannot be found (Campbell and Lack 1985). Observations carried out in platform nests showed that the abundance of invertebrates in nests yearly reused by birds, with additional building material added to the nest structure, was higher than in one-season nests (Błoszyk et al. 2005, 2009; Gwiazdowicz et al. 2005, 2006).

Thus, the nest characteristics, such as shape, building material (Pilskog et al. 2014; Gwiazdowicz et al. 2022), annual use or reuse (Gwiazdowicz et al. 1999; Błoszyk et al. 2009), and nest location (Ambros et al. 1992) influence the composition and abundance of mite fauna. When birds nest on the ground (Campbell and Lack 1985), they provide an easily available area to be inhabited by mites, especially by mite species associated with litter (Fenďa and Schniererová 2005). Such nests may be colonized by free-living or nidicolous mites but also by ectosymbiotic mite species including parasites, which are, at least seasonally, dependent on the presence of their bird host (Coulson et al. 2009). Other important factors affecting mite community composition in bird nests are the geographical region and climate. For example, in nests of snow buntings [*Plectrophenax nivalis* (L.)], located in the Arctic region (Spitsbergen, Svalbard), mainly females of the mite *Dermanyssus hirundinis* (Hermann) occur in large densities, whereas in other regions, nests of this bird species are inhabited by a greater diversity of mite taxa (Gwiazdowicz et al. 2012).

One of the bird species that builds nests exclusively on the ground is the wood warbler, *Phylloscopus sibilatrix* (Bechstein). This passerine, belonging to the family Phylloscopidae, is a small (~ 10 g) insectivorous, migratory songbird, with breeding grounds spanning northern and temperate Europe, as well as Central Asia (Cramp 1992). It is assumed to be a common bird, but in recent years scientists have observed declining trends in some regions, although the reasons of these trends are not sufficiently understood (Mallord et al. 2012a, b; Vickery et al. 2014; Mallord et al. 2016, 2017). The wood warbler is a forest-dwelling species, inhabiting mainly deciduous and mixed forests. Nests are dome-shaped, have a horizontally-oriented entrance, and are concealed usually among low herb vegetation in shady places, often near fallen branches or logs. Nests are constructed each season from plant materials, with the external layer of the nest made of decayed tree leaves or grasses, and mites have an opportunity to inhabit the nests at the beginning of the breeding season (Wesołowski 1985). Mites may colonize the nest directly from the ground but they can also be transported with nest-building material. So far, studies on wood warbler ecology have mainly focused on migration, breeding habitat selection and nest predation (Bellamy et al. 2018; Tøttrup et al. 2018). The nest fauna of this bird species has not been studied extensively. Some studies show that ants are frequently present in wood warbler nests (Maziarz et al. 2018), which can be associated with the thermal activity of the birds warming their nests (Maziarz et al. 2020, 2021). Among mites, only the Uropodina and Crotonioidea inhabiting wood warbler nests have been investigated so far (Napierała et al. 2021).

In this study we expand the knowledge on mite species colonizing wood warbler nests, taking into consideration all mite taxa, belonging to superorders Parasitiformes and Acariformes, found in nests during comprehensive sampling. Such knowledge is crucial to recognize the capability of mites to colonize ephemeral microhabitats that are available only for a few months every year. Such research is an essential foundation for studying intraspecific (mites-mites) and interspecific (mites-mites, mites-birds) interactions that may influence mite presence and abundance, and for explaining the role of 1-year nests as microhabitats for mites. Our aims were to (i) provide a comprehensive report on mite species inhabiting wood warbler nests, and (ii) assess infestation parameters (prevalence, intensity, and abundance) for mite species and higher taxa, to increase faunistic and ecological knowledge regarding these specific microhabitats.

## Materials and methods

### Study area

The study was conducted in the Wielkopolska National Park (WNP) in western Poland (52°13ʹ39.4ʹʹ–52°19ʹ08.0ʹʹ N, 16°32ʹ00.0ʹʹ–16°46ʹ08.0ʹʹ E). WNP covers an area of 75.84 km^2^, dominated by forests (61%) and farmland (ca. 30%) with a mosaic of fields, meadows, and wastelands. Forest habitats consist mainly of mixed forests dominated by pedunculate oak, *Quercus robur* L., sessile oak, *Quercus petraea* (Matt.) Liebl, or Scots pine, *Pinus sylvestris* L. In the WNP, wood warblers are found predominantly in mature oak-dominated and mixed oak-pine forests with a closed canopy, intermediate herb-layer cover, and bushes or trees branched in the lower stem area serving as song-posts (Szymkowiak et al. 2016, 2017; Szymkowiak and Kuczyński 2017).

### Collection of wood warbler nests

In 2013, territorial wood warblers were monitored in the WPN from mid-April to early July for a different study (see Szymkowiak et al. 2016 for details). After fledging or nest failure, we carefully collected the whole nest, placed it in a plastic string bag, and brought it to the laboratory for further processing. Nests were of sizes typical for this bird species (ca. 15 cm diameter). If the nest structure was damaged by a predator (eight incidents), all material that could be unambiguously assigned as nest remnants was collected. In total, we analyzed 45 wood warbler nests. In the laboratory, the microarthropod fauna was extracted using Berlese funnels with a mesh size of approximately 2 mm, under 40 W light bulbs, into 96% alcohol for 5 days until the nests were completely dry.

### Preparation and identification of mite taxa

Samples containing mite specimens stored in alcohol were inspected under stereomicroscopes and specimens belonging to different orders were counted and collected to separate vials. Subsequently, specimens were prepared for taxonomic identification to species by mounting on slides according to methods specific to each taxonomic group. We counted individuals representing the following taxa: superorder, order, suborder, cohort (in the case of Mesostigmata), family and species according to the systematic classification of Lindquist et al. (2009). Both adult and juvenile forms were counted, and juveniles were identified to the species level whenever possible, i.e., for Mesostigmata and Prostigmata.

#### Mesostigmata

The Sejida and Gamasida were mounted on permanent (Hoyer’s medium) and semipermanent (lactic acid) slides. The specimens were counted and examined under a microscope (Zeiss Axioscop 2), and identified using Ghilarov and Bregetova (1977), Karg (1993) and Gwiazdowicz (2007, 2010). Specimens of the Uropodina were mounted on semipermanent slides (lactic acid and glycerin) under a stereomicroscope (Olympus SZX16) and light microscope (Olympus BX53), and identified with Hirschmann and Zirngiebl-Nicol (1961), Kramer (1882), Karg (1989), Błoszyk (1999) and Mašán (2001).

#### Trombidiformes

Mite specimens were mounted in Berlese medium on microscope slides and thereafter stored in 70% alcohol. Mounted specimens were examined with phase contrast microscopes (Zeiss Peraval Inter-FAKO and Olympus BX91 Nomarski), and identified with Kuznetzov (1978a, b), Zacharda (1980), Michocka (1987), Smiley (1992), Kaźmierski (1998a, b), Jesionowska (2010), Skvarla et al. (2014), Hernandes et al. (2016), Silva et al. (2016), and Kaźmierski et al. (2021).

#### Sarcoptiformes

Mite specimens were cleaned for 24 h in lactic acid at room temperature (ca. 25 °C), mounted on temporary cavity slides for identification, and thereafter stored in 70% ethanol in vials. Individuals were identified with a microscope (Olympus BX51), using the key and descriptions in Weigmann (2006). Species nomenclature followed Subías (2004, updated 2022), Weigmann (2006), and Niedbała (2008).

### Statistical analysis

We calculated infestation indices for higher taxa (superorders and orders) and for each mite species. The following parameters were used (following Bush et al. 1997; Skoracka and Kuczyński 2012):

- *prevalence* the percentage of nests infested, interpreted as the probability of finding the mite taxon in a random nest;
- *intensity* the mean number of mite specimens of a given mite taxon found in an infested nest, interpreted as population density in nests occupied by that taxon; and
- *abundance* the mean number of mite specimens of a given taxon found in all nests, expressed in the same units as intensity (no. of individuals per nest), but reflecting population density across all potentially available nests.

Confidence intervals (95% CI) for prevalence were calculated using the profile likelihood method. Confidence intervals for abundance and intensity were calculated using a bias-corrected and accelerated bootstrap (Efron and Tibshirani 1993). All computations were made in R v.4.1 (R Foundation for Statistical Computing, Vienna, Austria, 2022).

## Results

**Fig. 1:**
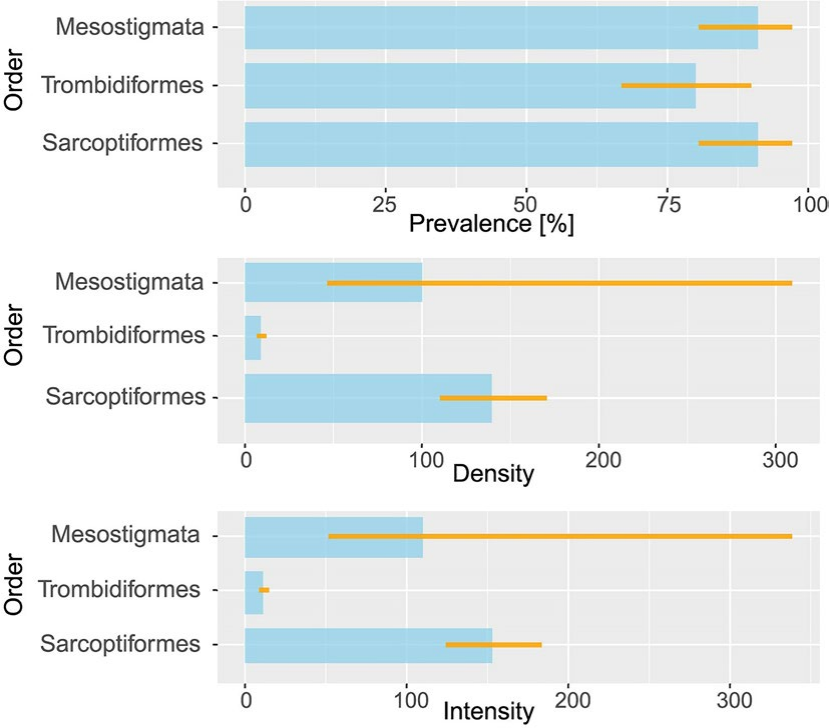
Nest infestation parameters calculated for mites grouped in orders: prevalence (top panel); intensity (middle panel) and abundance (bottom panel). Bars represent mean values and lines represent 95% confidence intervals around these means

The probability (*P*) of finding members of the Acariformes (represented by the Trombidiformes and Sarcoptiformes) as well as the intensity of nest infestation (*I*) and abundance (*A*) were similar to those of the Parasitiformes (represented by the Mesostigmata): *P*: 93.3% (95% CI 83.6–98.3%) vs. 91.1% (80.5–97.1%); *I*: 190.1 (153.5–226.8) vs. 110.0 (51.5–382.6); *A*: 177.4 (140.8–216.0) vs. 100.3 (46.5–312.3). Mites belonging to the orders Mesostigmata, Sarcoptiformes, and Trombidiformes had similar prevalence, whereas intensity and abundance of the Trombidiformes were much lower compared to the other two orders (for details see Fig. 1).

Parameters of infestation by mite species belonging to the Mesostigmata, Trombidiformes, and Sarcoptiformes are presented in Tables 1, 2 and 3. Among Mesostigmata, representatives of suborders Sejida and Monogynaspida were observed. The Sejida were represented solely by *Sejus togatus* C.L. Koch, whereas the Monogynaspida were represented by two cohorts: Uropodina (five species belonging to four families) and Gamasina (52 species belonging to 15 families) (Table 1).

**Table 1:** Nest infestation parameters (mean + 95% confidence interval in parentheses) calculated for mite species belonging to the Mesostigmata: prevalence (*P*, %), intensity (*I*, no. individuals per infested nest), and abundance (*A*, no. individuals per nest)

Suborder/infraorder	Family	Species	*k*	*P* [%]	*I*	*A*
Sejida	Sejidae	*Sejus togatus* C.L. Koch	3	6.7 (1.7–16.4)	1.0 (1.0–1.0)	0.1 (0.0–0.1)
Monogynaspida, Uropodina	Trachytidae	*Trachytes aegrota* (C.L. Koch)	30	66.7 (52.2–79.2)	11.9 (8.2–17.4)	7.9 (5.2–12.2)
	Trematuridae	*Nenteria breviunguiculata* (Willmann)	1	2.2 (0.2–9.4)	1 (NA–NA)	0.0 (0.0–0.1)
		*Oodinychus ovalis* (C.L. Koch)	33	73.3 (59.4–84.7)	16.9 (11.8–26.2)	12.4 (8.3–20.1)
	Urodinychidae	*Urodiaspis tecta* (Kramer)	17	37.8 (24.6–52.3)	5.6 (3.8–10.1)	2.1 (1.2–4.1)
	Uropodidae	*Olodiscus minima* (Kramer)	14	31.1 (18.9–45.4)	2.7 (1.8–4.8)	0.8 (0.4–1.7)
Monogynaspida, Gamasina	Ameroseiidae	*Epicriopsis horridus* (Kramer)	1	2.2 (0.2–9.4)	2 (NA–NA)	0.0 (0.0–0.1)
	Ascidae	*Arctoseius cetratus* (Sellnick)	1	2.2 (0.2–9.4)	1 (NA–NA)	0.0 (0.0–0.1)
		*Asca aphidioides* (L.)	9	20.0 (10.2–33.2)	3.2 (2.4–3.8)	0.6 (0.3–1.1)
		*Gamasellodes bicolor* (Berlese)	18	40.0 (26.5–54.6)	4.6 (3.4–5.8)	1.8 (1.1–2.8)
		*Leioseius elongatus* Evans	1	2.2 (0.2–9.4)	1 (NA–NA)	0.0 (0.0–0.1)
	Blattisociidae	*Lasioseius confusus* Evans	2	4.4 (0.8–13.1)	2.0 (2.0–2.0)	0.1 (0.0–0.2)
	Digamasellidae	*Dendrolaelaps* sp.	1	2.2 (0.2–9.4)	1 (NA–NA)	0.0 (0.0–0.1)
	Eviphididae	*Alliphis halleri* (G. et R. Canestrini)	16	35.6 (22.7–50.1)	4.7 (3.2–9.4)	1.7 (0.9–3.5)
		*Eviphis ostrinus* (C.L. Koch)	20	44.4 (30.5–59.0)	6.5 (3.9–11.2)	2.9 (1.6–5.6)
	Laelapidae	*Cosmolaelaps cuneifer* (Michael)	1	2.2 (0.2–9.4)	3 (NA–NA)	0.1 (0.0–0.2)
		*Cosmolaelaps vacua* (Michael)	4	8.9 (2.9–19.5)	1.5 (1.0–1.8)	0.1 (0.0–0.3)
		*Eulaelaps stabularis* (C.L. Koch)	2	4.4 (0.8–13.1)	1.0 (1.0–1.0)	0.0 (0.0–0.1)
		*Gaeolaelaps aculeifer* (Canestrini)	3	6.7 (1.7–16.4)	1.0 (1.0–1.0)	0.1 (0.0–0.1)
		*Gaeolaelaps brevipilis* Hirschmann et al.	1	2.2 (0.2–9.4)	1 (NA–NA)	0.0 (0.0–0.1)
		*Gaeolaelaps praesternalis* (Willmann)	3	6.7 (1.7–16.4)	2.0 (1.0–3.0)	0.1 (0.0–0.5)
		*Haemogamasus nidi* Michael	2	4.4 (0.8–13.1)	2.5 (2.0–2.5)	0.1 (0.0–0.3)
	Macrochelidae	*Geholaspis longispinosus* (Kramer)	7	15.6 (7.0–27.9)	3.4 (1.7–5.7)	0.5 (0.2–1.3)
		*Macrocheles glaber* (Müller)	4	8.9 (2.9–19.5)	2.0 (1.0–2.5)	0.2 (0.0–0.4)
		*Macrocheles montanus* (Willmann)	1	2.2 (0.2–9.4)	1 (NA–NA)	0.0 (0.0–0.1)
		*Macrocheles tridentinus* G. et R. Canestrini	1	2.2 (0.2–9.4)	1 (NA–NA)	0.0 (0.0–0.1)
		*Macrocheles* sp*.*	3	6.7 (1.7–16.4)	1.3 (1.0–1.7)	0.1 (0.0–0.2)
	Macronyssidae	*Ornithonyssus sylviarum* (Canestrini et Fanzago)	11	24.4 (13.5–38.2)	3.3 (2.1–5.2)	0.8 (0.4–1.6)
		*Ornithonyssus* sp*.*	1	2.2 (0.2–9.4)	2294 (NA–NA)	51.0 (0.0–152.9)
	Melicharidae	*Proctolaelaps juradeus* (Schweizer)	2	4.4 (0.8–13.1)	1.0 (1.0–1.0)	0.0 (0.0–0.1)
		*Proctolaelaps pygmaeus* (Müller)	8	17.8 (8.6–30.6)	4.4 (2.3–9.5)	0.8 (0.3–2.1)
	Ologamasidae	*Cyrtolaelaps mucronatus* (G. et R. Canestrini)	1	2.2 (0.2–9.4)	1 (NA–NA)	0.0 (0.0–0.1)
		*Euryparasitus emarginatus* C.L. Koch	1	2.2 (0.2–9.4)	1 (NA–NA)	0.0 (0.0–0.1)
	Pachylaelapidae	*Olopachys suecicus* Sellnick	3	6.7 (1.7–16.4)	2.3 (1.0–3.0)	0.2 (0.0–0.4)
		*Pachylaelaps furcifer* Oudemans	1	2.2 (0.2–9.4)	4 (NA–NA)	0.1 (0.0–0.3)
		*Pachylaelaps siculus* Berlese	2	4.4 (0.8–13.1)	1.5 (1.0–1.5)	0.1 (0.0–0.2)
	Parasitidae	*Cornigamasus lunaris* (Berlese)	2	4.4 (0.8–13.1)	1.0 (1.0–1.0)	0.0 (0.0–0.1)
		*Holoparasitus calcartaus* (Berlese)	18	40.0 (26.5–54.6)	3.1 (2.2–5.0)	1.2 (0.7–2.2)
		*Leptogamasus suecicus* Trägårdh	1	2.2 (0.2–9.4)	1 (NA–NA)	0.0 (0.0–0.1)
		*Paragamasus brevipes* (Berlese)	3	6.7 (1.7–16.4)	2.0 (1.0–3.0)	0.1 (0.0–0.5)
		*Paragamasus misellus* (Berlese)	10	22.2 (11.8–35.7)	6.4 (3.7–9.8)	1.4 (0.6–2.9)
		*Paragamasus runciger* (Berlese)	2	4.4 (0.8–13.1)	1.5 (1.0–1.5)	0.1 (0.0–0.2)
		*Paragamasus vagabundus* (Karg)	10	22.2 (11.8–35.7)	3.9 (2.2–7.4)	0.9 (0.4–2.0)
		*Parasitus fimetorum* (Berlese)	3	6.7 (1.7–16.4)	1.3 (1.0–1.7)	0.1 (0.0–0.2)
		*Pergamasus brevicornis* Berlese	4	8.9 (2.9–19.5)	1.5 (1.0–2.0)	0.1 (0.0–0.4)
		*Pergamasus crassipes* (L.)	1	2.2 (0.2–9.4)	1 (NA–NA)	0.0 (0.0–0.1)
		*Pergamasus mediocris* Berlese	6	13.3 (5.5–25.2)	4.7 (2.5–9.3)	0.6 (0.2–1.7)
		*Pergamasus norvegicus* (Berlese)	2	4.4 (0.8–13.1)	1.0 (1.0–1.0)	0.0 (0.0–0.1)
		*Vulgarogamasus kraepelini* (Berlese)	14	31.1 (18.9–45.4)	3.1 (2.4–3.9)	1.0 (0.5–1.5)
		*Vulgarogamasus remberti* (Oudemans)	1	2.2 (0.2–9.4)	1 (NA–NA)	0.0 (0.0–0.1)
	Phytoseiidae	*Amblyseius* sp*.*	8	17.8 (8.6–30.6)	1.4 (1.0–1.6)	0.2 (0.1–0.4)
	Veigaiidae	*Veigaia cervus* (Kramer)	15	33.3 (20.8–47.8)	5.5 (3.5–8.3)	1.8 (1.0–3.3)
		*Veigaia kochi* (Trägårdh)	8	17.8 (8.6–30.6)	2.0 (1.3–3.1)	0.4 (0.1–0.8)
		*Veigaia nemorensis* (C.L. Koch)	23	51.1 (36.8–65.3)	5.4 (4.3–6.6)	2.8 (1.9–3.9)
	Zerconidae	*Parazercon radiatus* (Berlese)	1	2.2 (0.2–9.4)	1 (NA–NA)	0.0 (0.0–0.1)
		*Prozercon kochi* Sellnick	14	31.1 (18.9–45.4)	5.1 (2.9–9.2)	1.6 (0.8–3.2)
		*Zercon peltatus peltatus* C.L. Koch	17	37.8 (24.6–52.3)	7.1 (4.1–13.5)	2.7 (1.4–5.7)
		*Zercon triangularis* C.L. Koch	3	6.7 (1.7–16.4)	1.0 (1.0–1.0)	0.1 (0.0–0.1)

*k* number of nests of the 45 nests examined infested by a given taxon. *NA* not applicable (the parameter cannot be calculated for k = 1)

**Table 2:** Nest infestation parameters (mean + 95% confidence interval in parentheses) calculated for mite species belonging to the Trombidiformes (represented by the Prostigmata): prevalence (*P*, %), intensity (*I*, no. individuals per infested nest), and abundance (*A*, no. individuals per nest)

Family	Species	*k*	*P* [%]	*I*	*A*
Bdellidae	*Bdella dispar* (C.L. Koch)	3	6.7 (1.7–16.4)	2.3 (1.0–3.7)	0.2 (0.0–0.6)
	*Bdella strandi* (Berlese)	1	2.2 (0.2–9.4)	1 (NA–NA)	0.0 (0.0–0.1)
	*Biscirus silvaticus* (Kramer)	1	2.2 (0.2–9.4)	3 (NA–NA)	0.1 (0.0–0.2)
	*Cyta latirostris* (Hermann)	1	2.2 (0.2–9.4)	1 (NA–NA)	0.0 (0.0–0.1)
	*Odontoscirus atyeoi* Michocka	2	4.4 (0.8–13.1)	1.0 (1.0–1.0)	0.0 (0.0–0.1)
	*Odontoscirus iota* (Atyeo)	1	2.2 (0.2–9.4)	1 (NA–NA)	0.0 (0.0–0.1)
	*Spinidbella reducta* Thor	2	4.4 (0.8–13.1)	1.5 (1.0–1.5)	0.1 (0.0–0.2)
Cheyletidae	*Cheletogenes ornatus* (Canestrini et Fanzago)	1	2.2 (0.2–9.4)	1 (NA–NA)	0.0 (0.0–0.1)
Cocceupodidae	*Cocceupodes mollicellus* (C.L. Koch)	2	4.4 (0.8–13.1)	1.0 (1.0–1.0)	0.0 (0.0–0.1)
	*Cocceupodes stellatus* Strandtmann et Prasse	4	8.9 (2.9–19.5)	4.8 (1.5–10.5)	0.4 (0.1–1.6)
	*Filieupodes filiformis* Jesionowska	3	6.7 (1.7–16.4)	1.0 (1.0–1.0)	0.1 (0.0–0.2)
	*Filieupodes filistellatus* Jesionowska	2	4.4 (0.8–13.1)	3.0 (1.0–3.0)	0.1 (0.0–0.6)
	*Filieupodes* sp.	1	2.2 (0.2–9.4)	1 (NA–NA)	0.0 (0.0–0.1)
	*Linopodes* sp. 1	5	11.1 (4.1–22.4)	4.0 (1.4–6.4)	0.4 (0.1–1.2)
	*Linopodes* sp. 2	10	22.2 (11.8–35.7)	1.6 (1.1–2.1)	0.4 (0.2–0.6)
Cunaxidae	*Armascirus fendai* Kaluz et Vrabec	2	4.4 (0.8–13.1)	1.5 (1.0–1.5)	0.1 (0.0–0.2)
	*Cunaxa setirostris* (Hermann)	14	31.1 (18.9–45.4)	2.1 (1.4–3.3)	0.6 (0.3–1.2)
	*Cunaxa* sp. nov.	4	8.9 (2.9–19.5)	1.3 (1.0–1.5)	0.1 (0.0–0.2)
	*Cunaxa womersleyi* Baker et Hoffmann	1	2.2 (0.2–9.4)	1 (NA–NA)	0.0 (0.0–0.1)
	*Cunaxoides croceus* (C.L. Koch)	1	2.2 (0.2–9.4)	1 (NA–NA)	0.0 (0.0–0.1)
	*Cunaxoides kielczewskii* Michocka	1	2.2 (0.2–9.4)	1 (NA–NA)	0.0 (0.0–0.1)
Ereynetidae	*Ereynetes* (*Ereynetes*) *galeatus* Berlese	3	6.7 (1.7–16.4)	2.0 (1.0–3.0)	0.1 (0.0–0.4)
	*Ereynetes* (*Gymnereynetes*) *exilis* Fain et Prasse	1	2.2 (0.2–9.4)	3 (NA–NA)	0.1 (0.0–0.2)
	*Ereynetes* (*Gymnereynetes*) sp. 1	3	6.7 (1.7–16.4)	1.7 (1.0–2.0)	0.1 (0.0–0.3)
	*Ereynetes* (*Gymnereynetes*) sp. 2	1	2.2 (0.2–9.4)	1 (NA–NA)	0.0 (0.0–0.1)
Erythraeidae	*Abrolophus miniatus* (Hermann)	1	2.2 (0.2–9.4)	1 (NA–NA)	0.0 (0.0–0.1)
Eupodidae	*Eupodes* sp. 1	3	6.7 (1.7–16.4)	2.0 (1.0–2.7)	0.1 (0.0–0.4)
	*Eupodes* sp. 2	4	8.9 (2.9–19.5)	2.0 (1.0–2.5)	0.2 (0.0–0.4)
	*Eupodes* sp. 3	2	4.4 (0.8–13.1)	1.0 (1.0–1.0)	0.0 (0.0–0.1)
	*Eupodes* sp. 4	3	6.7 (1.7–16.4)	1.3 (1.0–1.7)	0.1 (0.0–0.2)
	*Eupodes voxencollinus* Thor	15	33.3 (20.8–47.8)	1.8 (1.3–2.5)	0.6 (0.3–1.0)
	*Protereunetes* sp.	1	2.2 (0.2–9.4)	1 (NA–NA)	0.0 (0.0–0.1)
Iolinidae	*Microtydeus beltrani* Baker	4	8.9 (2.9–19.5)	2.5 (1.0–4.8)	0.2 (0.0–0.7)
	*Microtydeus subtilis* (C.L. Koch)	1	2.2 (0.2–9.4)	2 (NA–NA)	0.0 (0.0–0.1)
	*Tydaeolus tenuiclaviger* (Thor)	1	2.2 (0.2–9.4)	1 (NA–NA)	0.0 (0.0–0.1)
Microtrombidiidae	*Dactylothrombidium pulcherrimum* (Haller)	2	4.4 (0.8–13.1)	1.0 (1.0–1.0)	0.0 (0.0–0.1)
Pygmephoroidae	*Bakerdania* sp. 1	1	2.2 (0.2–9.4)	3 (NA–NA)	0.1 (0.0–0.2)
	*Bakerdania* sp. 2	1	2.2 (0.2–9.4)	1 (NA–NA)	0.0 (0.0–0.1)
	*Bakerdania* sp. 3	1	2.2 (0.2–9.4)	1 (NA–NA)	0.0 (0.0–0.1)
	*Bakerdania* sp. 4	1	2.2 (0.2–9.4)	1 (NA–NA)	0.0 (0.0–0.1)
Rhagididae	*Poecilophysis* sp. 1	5	11.1 (4.1–22.4)	1.6 (1.0–2.2)	0.2 (0.0–0.4)
	*Poecilophysis* sp. 2	2	4.4 (0.8–13.1)	1.0 (1.0–1.0)	0.0 (0.0–0.1)
Stigmaeidae	*Cheylostigmaeus* sp.	1	2.2 (0.2–9.4)	1 (NA–NA)	0.0 (0.0–0.1)
	*Eustigmaeus formosus* Kaźmierski et Dończyk	4	8.9 (2.9–19.5)	3.0 (1.5–5.3)	0.3 (0.1–0.7)
	*Eustigmaeus lacuna* (Summers)	1	2.2 (0.2–9.4)	1 (NA–NA)	0.0 (0.0–0.1)
	*Eustigmaeus myrtea* (Chaudhri)	9	20.0 (10.2–33.2)	2.8 (1.3–6.4)	0.6 (0.2–1.6)
	*Eustigmaeus segnis* (C.L. Koch)	3	6.7 (1.7–16.4)	1.0 (1.0–1.0)	0.1 (0.0–0.1)
	Gen. nov. sp. nov.	1	2.2 (0.2–9.4)	7 (NA–NA)	0.2 (0.0–0.5)
	*Mediolata* sp. nov.	1	2.2 (0.2–9.4)	1 (NA–NA)	0.0 (0.0–0.1)
	*Stigmaeus clitellus* Summers	1	2.2 (0.2–9.4)	1 (NA–NA)	0.0 (0.0–0.1)
	*Stigmaeus corticeus* Kuznetzov et Wainstein	1	2.2 (0.2–9.4)	4 (NA–NA)	0.1 (0.0–0.3)
	*Stigmaeus insectus* Willmann	2	4.4 (0.8–13.1)	1.0 (1.0–1.0)	0.0 (0.0–0.1)
	*Stigmaeus longipilis* (Canestrini)	16	35.6 (22.7–50.1)	2.5 (1.7–3.8)	0.9 (0.5–1.5)
	*Stigmaeus longisetis* Wood	1	2.2 (0.2–9.4)	2 (NA–NA)	0.0 (0.0–0.1)
	*Stigmaeus obtectus* Summers	1	2.2 (0.2–9.4)	2 (NA–NA)	0.0 (0.0–0.1)
	*Stigmaeus pilatus* Kuznetzov	11	24.4 (13.5–38.2)	1.8 (1.3–2.4)	0.4 (0.2–0.8)
	*Stigmaeus rhodomelas* Berlese	4	8.9 (2.9–19.5)	1.5 (1.0–1.8)	0.1 (0.0–0.3)
	*Stigmaeus sphagneti* (Hull)	22	48.9 (34.7–63.2)	2.1 (1.5–3.0)	1.0 (0.7–1.6)
	*Stigmaeus* sp. nov.	1	2.2 (0.2–9.4)	3 (NA–NA)	0.1 (0.0–0.2)
Tarsonemidae	*Tarsonemus limbatus* (Hammen)	1	2.2 (0.2–9.4)	1 (NA–NA)	0.0 (0.0–0.1)
Tydeidae	*Lorryia brevicula* (C.L. Koch)	1	2.2 (0.2–9.4)	1 (NA–NA)	0.0 (0.0–0.1)
	*Lorryia subularoides* Kaźmierski	1	2.2 (0.2–9.4)	1 (NA–NA)	0.0 (0.0–0.1)
	*Lorryia superba* Oudemans	1	2.2 (0.2–9.4)	1 (NA–NA)	0.0 (0.0–0.1)
	*Lorryia* sp.	1	2.2 (0.2–9.4)	1 (NA–NA)	0.0 (0.0–0.1)
	*Tydeus clavimaculatus* Kaźmierski	2	4.4 (0.8–13.1)	1.0 (1.0–1.0)	0.0 (0.0–0.1)

*k* number of nests of the 45 nests examined infested by a given taxon. *NA* not applicable (parameter cannot be calculated for k = 1)

**Table 3:** Nest infestation parameters (mean + 95% confidence interval in parentheses) calculated for mite species belonging to the Sarcoptiformes: prevalence (*P*, %), intensity (*I*, no. individuals per infested nest), and abundance (*A*, no. individuals per nest)

Suborder	Family	Species	k	*P* [%]	*I*	*A*
Oribatida	Achipteridae	*Achipteria coleoptrata* (L.)	8	17.8 (8.6–30.6)	11.4 (5.4–21.6)	2.0 (0.7–5.0)
		*Achipteria nitens* (Nicolet)	4	8.9 (2.9–19.5)	3.8 (2.0–6.8)	0.3 (0.1–1.0)
	Autognetidae	*Autogneta longilamellata* (Michael)	9	20.0 (10.2–33.2)	4.0 (2.9–5.3)	0.8 (0.4–1.5)
	Camisiidae	*Platynothrus peltifer* (C.L. Koch)	31	68.9 (54.6–81.1)	11.1 (6.9–17.8)	7.7 (4.6–12.7)
	Carabodidae	*Carabodes areolatus *Berlese	13	28.9 (17.1–43.1)	3.0 (1.9–4.7)	0.9 (0.4–1.6)
		*Carabodes coriaceus* C.L. Koch	8	17.8 (8.6–30.6)	4.8 (2.4–8.4)	0.8 (0.3–1.9)
		*Carabodes labyrinthicus* (Michael)	7	15.6 (7.0–27.9)	1.3 (1.0–1.6)	0.2 (0.1–0.4)
		*Carabodes ornatus* Štorkán	19	42.2 (28.5–56.8)	3.1 (2.3–4.1)	1.3 (0.8–2.0)
		*Carabodes reticulatus* Berlese	19	42.2 (28.5–56.8)	4.4 (3.1–7.4)	1.8 (1.1–3.3)
	Cepheidae	*Cepheus cepheiformis* (Nicolet)	9	20.0 (10.2–33.2)	1.4 (1.1–1.7)	0.3 (0.1–0.5)
	Ceratozetidae	*Trichoribates novus* (Sellnick)	10	22.2 (11.8–35.7)	2.4 (1.5–4.4)	0.5 (0.2–1.2)
	Chamobatidae	*Chamobates subglobulus* (Oudemans)	5	11.1 (4.1–22.4)	5.2 (2.4–8.0)	0.6 (0.2–1.5)
		*Chamobates voigtsi* (Oudemans)	8	17.8 (8.6–30.6)	5.9 (3.8–10.3)	1.0 (0.5–2.3)
	Crotoniidae	*Camisia spinifer* (C.L. Koch)	4	8.9 (2.9–19.5)	1.8 (1.0–2.5)	0.2 (0.0–0.4)
	Damaeidae	*Belba corynopus* (Hermann)	19	42.2 (28.5–56.8)	2.7 (2.1–3.7)	1.2 (0.7–1.8)
		*Damaeus auritus* C.L. Koch	11	24.4 (13.5–38.2)	2.6 (1.6–4.5)	0.6 (0.3–1.3)
		*Damaeus clavipes* (Hermann)	4	8.9 (2.9–19.5)	1.3 (1.0–1.5)	0.1 (0.0–0.2)
		*Damaeus onustus* C.L. Koch	21	46.7 (32.6–61.1)	3.7 (2.7–4.9)	1.7 (1.1–2.6)
		*Metabelba papillipes* (Nicolet)	13	28.9 (17.1–43.1)	5.6 (3.8–8.1)	1.6 (0.8–2.8)
		*Metabelba pulverosa* Strenzke	35	77.8 (64.3–88.2)	40.9 (32.2–52.5)	31.8 (23.6–42.4)
	Eniochthoniidae	*Eniochthonius minutissimus* (Berlese)	2	4.4 (0.8–13.1)	3.0 (1.0–3.0)	0.1 (0.0–0.5)
	Eremaeidae	*Eueremaeus oblongus* (C.L. Koch)	4	8.9 (2.9–19.5)	14.8 (5.0–32.8)	1.3 (0.2–5.1)
	Euphthiracaridae	*Acrotritia ardua* (C.L. Koch)	1	2.2 (0.2–9.4)	5 (NA–NA)	0.1 (0.0–0.3)
		*Acrotritia duplicata* (Grandjean)	10	22.2 (11.8–35.7)	10.4 (4.9–26.8)	2.3 (0.9–6.9)
		*Euphthiracarus cribrarius* (Berlese)	6	13.3 (5.5–25.2)	1.7 (1.0–2.3)	0.2 (0.1–0.5)
		*Microtritia minima* (Berlese)	9	20.0 (10.2–33.2)	2.1 (1.3–3.0)	0.4 (0.2–0.8)
	Euzetidae	*Euzetes globulus* (Nicolet)	12	26.7 (15.3–40.6)	5.7 (2.8–13.9)	1.5 (0.6–4.4)
	Galumnidae	*Acrogalumna longipluma* (Berlese)	6	13.3 (5.5–25.2)	2.7 (1.7–3.7)	0.4 (0.1–0.8)
		*Galumna lanceata* Oudemans	6	13.3 (5.5–25.2)	2.3 (1.0–4.0)	0.3 (0.1–0.8)
		*Galumna obvia* (Berlese)	9	20.0 (10.2–33.2)	3.7 (2.2–5.6)	0.7 (0.3–1.5)
		*Pergalumna nervosa* (Berlese)	12	26.7 (15.3–40.6)	3.8 (2.7–5.8)	1.0 (0.5–1.8)
	Liacaridae	*Adoristes ovatus* (C.L. Koch)	10	22.2 (11.8–35.7)	3.5 (2.3–4.9)	0.8 (0.4–1.4)
		*Liacarus coracinus* (C.L. Koch)	10	22.2 (11.8–35.7)	4.3 (2.3–9.0)	1.0 (0.4–2.4)
		*Xenillus tegeocranus* (Hermann)	3	6.7 (1.7–16.4)	3.7 (1.0–5.7)	0.2 (0.0–0.9)
	Malaconothridae	*Malaconothrus monodactylus* (Michael)	2	4.4 (0.8–13.1)	1.5 (1.0–1.5)	0.1 (0.0–0.2)
	Micreremidae	*Micreremus brevipes* (Michael)	10	22.2 (11.8–35.7)	5.1 (3.2–7.6)	1.1 (0.5–2.2)
	Nanhermanniidae	*Nanhermannia nana* (Nicolet)	21	46.7 (32.6–61.1)	4.8 (2.6–10.9)	2.2 (1.1–5.4)
	Neoliodidae	*Poroliodes farinosus* (C.L. Koch)	9	20.0 (10.2–33.2)	2.9 (1.3–7.0)	0.6 (0.2–1.7)
	Nothridae	*Nothrus silvestris* Nicolet	15	33.3 (20.8–47.8)	4.5 (1.9–13.7)	1.5 (0.6–5.0)
	Oppiidae	*Disorrhina ornata* (Oudemans)	23	51.1 (36.8–65.3)	9.6 (6.7–14.7)	4.9 (3.1–8.1)
		*Oppia nitens* C.L. Koch	11	24.4 (13.5–38.2)	5.0 (3.0–7.8)	1.2 (0.6–2.4)
		*Oppiella falcata* (Paoli)	17	37.8 (24.6–52.3)	6.9 (3.9–14.0)	2.6 (1.3–5.8)
		*Oppiella nova* (Oudemans)	21	46.7 (32.6–61.1)	9.0 (6.1–13.0)	4.2 (2.5–6.8)
		*Oppiella subpectinata* (Oudemans)	9	20.0 (10.2–33.2)	4.7 (2.0–12.0)	0.9 (0.3–3.0)
		*Oppiella unicarinata* (Paoli)	18	40.0 (26.5–54.6)	6.7 (4.1–10.6)	2.7 (1.4–4.7)
		*Ramusella insculpta* (Paoli)	18	40.0 (26.5–54.6)	7.4 (4.9–10.7)	3.0 (1.7–4.9)
	Oribatellidae	*Oribatella quadricornuta* Bernini	5	11.1 (4.1–22.4)	1.8 (1.0–3.0)	0.2 (0.0–0.5)
		*Oribatidaella brevipila* Michael	9	20.0 (10.2–33.2)	5.4 (3.2–8.9)	1.1 (0.4–2.3)
	Oribatulidae	*Oribatula tibialis* (Nicolet)	9	20.0 (10.2–33.2)	5.9 (3.2–11.3)	1.2 (0.5–2.8)
		*Zygoribatula exilis* (Nicolet)	8	17.8 (8.6–30.6)	4.3 (2.5–6.3)	0.8 (0.3–1.6)
	Pelopiidae	*Ceratoppia bipilis* (Hermann)	7	15.6 (7.0–27.9)	2.7 (1.4–4.7)	0.4 (0.2–1.0)
		*Ceratoppia quadridentata* (Haller)	4	8.9 (2.9–19.5)	1.3 (1.0–1.5)	0.1 (0.0–0.2)
	Phenopelopidae	*Eupelops hirtus* (Berlese)	15	33.3 (20.8–47.8)	3.6 (2.3–5.3)	1.2 (0.6–2.1)
		*Eupelops occultus* (C.L. Koch)	13	28.9 (17.1–43.1)	4.3 (2.2–8.2)	1.2 (0.5–2.8)
		*Eupelops torulosus* (C.L. Koch)	6	13.3 (5.5–25.2)	2.5 (1.0–5.2)	0.3 (0.1–1.0)
		*Peloptulus phaenotus* (C.L. Koch)	7	15.6 (7.0–27.9)	2.0 (1.3–2.6)	0.3 (0.1–0.6)
	Phthiracaridae	*Phthiracarus bryobius* Jacot	5	11.1 (4.1–22.4)	4.0 (2.0–6.2)	0.4 (0.1–1.2)
		*Phthiracarus crinitus* (C.L. Koch)	1	2.2 (0.2–9.4)	2 (NA–NA)	0.0 (0.0–0.1)
		*Phthiracarus laevigatus* (C.L. Koch)	5	11.1 (4.1–22.4)	2.0 (1.0–2.6)	0.2 (0.1–0.5)
		*Phthiracarus longulus* (C.L. Koch)	37	82.2 (69.4–91.4)	4.8 (3.6–6.3)	3.9 (2.9–5.3)
		*Phthiracarus nitens* (Nicolet)	12	26.7 (15.3–40.6)	5.3 (3.5–7.0)	1.4 (0.7–2.4)
	Quadroppiidae	*Quadroppia quadricarinata* (Michael)	7	15.6 (7.0–27.9)	2.4 (1.4–3.3)	0.4 (0.1–0.8)
	Scheloribatidae	*Scheloribates initialis* (Berlese)	18	40.0 (26.5–54.6)	4.1 (3.0–5.7)	1.6 (1.0–2.6)
		*Scheloribates laevigatus* (C.L. Koch)	16	35.6 (22.7–50.1)	5.7 (4.3–7.8)	2.0 (1.2–3.3)
	Steganacaridae	*Steganacarus carinatus* (C.L. Koch)	30	66.7 (52.2–79.2)	27.9 (20.1–40.0)	18.6 (12.4–27.5)
		*Atropacarus striculus* (C.L. Koch)	2	4.4 (0.8–13.1)	5.0 (4.0–5.0)	0.2 (0.0–0.7)
	Suctobelbidae	*Suctobelbella subtrigona* (Oudemans)	3	6.7 (1.7–16.4)	5.7 (2.0–8.7)	0.4 (0.0–1.3)
		*Suctobelbella subcornigera* (Forsslund)	5	11.1 (4.1–22.4)	2.0 (1.0–3.0)	0.2 (0.1–0.5)
	Tectocepheidae	*Tectocepheus velatus* (Michael)	33	73.3 (59.4–84.7)	10.5 (7.4–18.3)	7.7 (5.2–13.5)
	Tenuialidae	*Hafenrefferia gilvipes* (C.L. Koch)	1	2.2 (0.2–9.4)	1 (NA–NA)	0.0 (0.0–0.1)
	Thyrisomidae	*Banksinoma lanceolata* (Michael)	16	35.6 (22.7–50.1)	4.4 (2.8–6.4)	1.6 (0.8–2.7)
	Trhypochthoniidae	*Trhypochthonius tectorum* (Berlese)	5	11.1 (4.1–22.4)	2.4 (1.0–4.4)	0.3 (0.1–0.8)
	Zetomimidae	*Heterozetes palustris* (Willmann)	15	33.3 (20.8–47.8)	7.2 (5.1–10.3)	2.4 (1.4–4.1)
		Juveniles	38	84.4 (72.1–93.0)	34.3 (26.3–44.1)	29.0 (21.7–38.0)
Endeostigmata	Alycidae	*Alycus* sp.	2	4.4 (0.8–13.1)	2.0 (2.0–2.0)	0.1 (0.0–0.2)
	Nanorchestidae	*Nanorchestets* sp.	1	2.2 (0.2–9.4)	1 (NA–NA)	0.0 (0.0–0.1)

*k* number of nests of the 45 nests examined infested by a given taxon. *NA* not applicable (the parameter cannot be calculated for k = 1)

The Trombidiformes were represented only by the suborder Prostigmata, which was characterized by high overall richness (65 species belonging to 14 families). The highest prevalence (> 20% up to almost 50%) was observed for *Stigmaeus sphagneti* (Hull) (22 nests), *Stigmaeus longipilis* (Canestrini) (16), *Eupodes voxencollinus* Thor (15), *Cunaxa setirostris* (Hermann) (14), *Stigmaeus pilatus* Kuznetsov (11), and *Linopodes* sp. 2 (10) (Table 2).

Within the Sarcoptiformes, 73 species belonging to 35 families of the suborder Oribatida and two species belonging to two families of the suborder Endeostigmata were recorded (Table 3).

## Discussion

Our extensive investigation of wood warbler nests resulted in the identification of a huge diversity of mites (198 species) occurring in this ephemeral, single-season microhabitat. Representatives of both mites’ phylogenetic lineages—the Parasitiformes and Acariformes—were present in the nests. Considering the order level, the probability of finding representatives belonging to the Mesostigmata, Sarcoptiformes, and Trombidiformes was high (prevalence ≥ 80%). However, the Trombidiformes, represented in our study only by the Prostigmata, achieved significantly lower levels of intensity and density, as compared to members of the other orders. This suggests that although the Prostigmata can easily and frequently enter wood warbler nests, their survival or reproduction may be restricted, e.g., due to unsuitable abiotic conditions or competition with representatives of other mite orders, which attain much higher densities. Another scenario is that the Prostigmata generally are less abundant than representatives of other taxa in the soil, from which they can move to the nests. For example, Dziuba (1966) showed that in Poland, regardless of the cultivated plant and soil structure, the Trombidiformes (including Prostigmata) were the least numerous group of soil mites, whereas the Mesostigmata dominated in soils with inferior (compact) structure and the Sarcoptiformes dominated in soils with better (granular) structure. The monitoring of soil mites in various agroecosystems and forest ecosystems in Kenya revealed that Prostigmata mites were less abundant than the Oribatida and Mesostigmata (Maribie et al. 2011). Similarly, in natural soil of Argentina the Oribatida and Mesostigmata were more numerous than the Prostigmata (Bedano et al. 2005). Hasegawa et al. (2013) analysed the soil communities of Mesostigmata, Prostigmata, and Oribatida in broad-leaved regeneration forests and conifer plantations in Japan, and demonstrated that oribatid mites dominated in terms of densities and species richness for both forest types.

Among mites belonging to the Mesostigmata we found representatives of the suborders Sejida and Monogynaspida. From the suborder Sejida, only *S. togatus* was recorded. It inhabits various microhabitats, such as forest litter, rotting wood, ant nests, and bark beetle feeding grounds (Gwiazdowicz 2010). Although it was previously found in nests of white-tailed eagle, *Haliaeetus albicilla* (L.), it is not considered to be closely associated with microhabitats of nest birds (Gwiazdowicz et al. 2005). Low nest infestation parameters of *S. togatus* in our study support the suggestion that this species is not typical for bird nests.

From the suborder Monogynaspida, representatives of the cohorts Uropodina and Gamasina were identified in the wood warbler nests. More than 50 species of Gamasina were recorded, most of which usually inhabit forest litter and rotting wood. Only a few species indicated in the analyzed material were previously found in bird nests. In this context, it is worth mentioning that the parasite *Ornithonyssus sylviarum* (Canestrini et Fanzago) has been found in nests of many bird species, e.g., barn swallow (*Hirundo rustica* L.), greenfinch (*Chloris chloris* L.), rook (*Corvus frugilegus* L.), or song thrush (*Turdus philomelos* Brehm) (Micherdziński 1980). This blood-sucking mite also affects various domestic birds, including chickens, ducks, pigeons, parrots, and canaries, and is increasingly found to cause problems in aviaries and the poultry industry (Knee and Proctor 2007; Murillo and Mullens 2017). In the material we analyzed, *O. sylviarum* occurred in 11 nests, so the probability of finding this species in wood warbler nests was > 24%. In addition, several species known from the nests of other bird species were also found in this study, but their numbers were low. For example, *Alliphis halleri* (G. et R. Canestrini) and *Parasitus fimetorum* (Berlese), occurring in huge numbers in the nests of *Haliaeetus albicilla* L. (Gwiazdowicz et al. 2006), were found sporadically in the analyzed material. Although the probability of finding *A. halleri* in wood warbler nests was > 35%, the intensity of infestation and density were very low. Therefore, most Gamasina found in this study are more characteristic for the soil environment and forest litter than for bird nests.

Representatives of the Uropodina can be found at all latitudes, except polar regions. They occur wherever organic matter accumulates, and inhabit diverse habitats from coastal and inland dunes to rocky grasslands at the highest elevations. Although they clearly prefer the litter and soil of different types of forest ecosystems, they are just as likely to inhabit ephemeral microhabitats (merocenoses), such as cavities, dead wood, anthills, bird and mammal nests, and feces (Błoszyk 1999). Some members of the Uropodina colonize both annual bird nests and perennial nests of birds of prey and storks (Błoszyk and Olszanowski 1985; Błoszyk et al. 2005, 2006, 2009, 2016). Among the nearly 150 species of the Uropodina in Poland, 28 were found to be closely associated with bird nests (Napierała and Błoszyk 2013). In this study, only five Uropodina species were found in wood warbler nests in the Wielkopolska National Park: four of them were relatively frequent (prevalence 31–73%), and two of them had quite high intensity of infestation and density, compared to other Mesostigmata species and species belonging to two other orders. However, none of these species was a typical nidicole; instead, all were representatives of soil fauna. A similar outcome has been reported by Napierała et al. (2021), who studied wood warbler nests in Białowieża Forest. Akin to Napierała et al. (2021), in our study *Oodinychus ovalis* (CL Koch) achieved the highest parameters of infestation, compared to other species found in wood warbler nests. This is probably due to the fact that it is a highly genetically polymorphic species and the most numerous representative of the Uropodina in Poland. It occurs as often in the soil as in deadwood merocenoses (Błoszyk et al. 2019). Additionally, the fauna of the Uropodina in our study was notably less diverse than the Uropodina fauna found in wood warbler nests in Białowieża Forest, which included 14 species (Napierała et al. 2021).

The order Trombidiformes (represented by the Prostigmata) is an ecologically heterogeneous group of mites. They are cosmopolitan and occur in a myriad of microhabitats, including nests of vertebrates. The mites also remarkably vary in food preference, feeding behavior, and biotic associations. Some of them are predators, others are scavengers (saprophagous), and others are phytophagous or parasitic. In our study the suborder Prostigmata was characterized by high prevalence and overall richness, but very low density and intensity of infestation. The highest probability of finding prostigmatid species in wood warbler nests was for *Stigmaeus sphagneti*, *S. longipilis*, *S. pilatus*, *Eupodes voxencollinus*, *Cunaxa setirostris*, and *Linopodes* sp. 2. These common species are known from various environments, but they prefer mosses and lichens, which are used by the wood warbler to build its nest. This may explain their high prevalence in nests – they were simply brought along with the building material. Their low density and intensity of infestation may result from unsuitable abiotic conditions or negative biotic interactions, e.g., competition. The other species were recorded from a few or even single nests, which may suggest that they were accidental. Among these accidental species, we found representatives of a new genus, which indicates that the Prostigmata fauna in the soil, litter or vegetation surrounding warbler nests still hides undescribed species.

The Oribatida (Sarcoptiformes) are an extremely diverse and dominant group of mites, mainly in the soil organic layer in temperate forest. They can be found wherever dead organic matter occurs, specifically in mosses, lichens, rock cavities, tree crowns, on herbaceous plants, in rotting wood, and in bird and mammal nests. Most of them are saprophagous and fungivorous (Walter and Proctor 2013). In our study we found 73 species of oribatids, some with relatively medium or high infestation parameters. Wood warbler nests are built directly on the ground from organic debris, grass, moss, and dry branches. Therefore, the presence of all these oribatid species was, most likely, accidental and associated with materials gathered by the birds for nest building. We found no species typical of bird nests nor any species which could have symbiotic relationships with birds. All the species we found are common in Poland, and most of them have a Palearctic distribution. Interestingly, the diversity of ptyctimous oribatids in wood warbler nests in our study (11 species) was much lower than that found in wood warbler nests in Białowieża Forest (20 species) (Wojciech Niedbała, unpubl. data)—an outcome similar to that of uropodine mites. We did not find the following species that were present in nests investigated in Białowieża Forest: *Mesoplophora pulchra* Sellnick, *Phthiracarus boresetosus* Jacot, *Phthiracarus clavatus* Parry, *Phthiracarus compressus* Jacot, *Phthiracarus globosus* C.L. Koch, *Steganacarus applicatus* (Sellnick), *Steganacarus magnus* (Nicolet), *Steganacarus spinosus* (Sellnick). This difference likely results from higher species richness of mites and more diverse habitats in Białowieża Forest, compared to Wielkopolska National Park.

The presence of invertebrates in nests may lead to the evolution of different relationships (e.g., commensal or mutualistic) between birds and invertebrate nest inhabitants. For example, ants are attracted to wood warbler nests by the heat generated by the host (Maziarz et al. 2020). The occurrence of mites in nests may also be the first step to parasitism on birds. For example, the prostigmatid Speleognathinae (Ereynetidae, Tydeoidea), which live as parasites in bird nostrils, likely originated from free-living ancestors inhabiting the nests (Kaźmierski et al. 2018). In another ereynetid subfamily, Ereynetinae, a tendency to parasitism is also observed: *Ricardoella limacum* (Schrank) lives in the mantle cavity of pulmonary snails (Turk and Phillips 1946; Karbarz-Wiktorowicz 1973), whereas *Hydranetes tropisternus* Kethley infects water beetles (Kethley 1971). Undoubtedly, the cohabitation of birds and nest-dwelling invertebrates may stimulate the evolution of interspecific interactions (Maziarz et al. 2020). Such relationships are poorly studied. A quantitative report, such as the present one, can be an important first step for such investigations, by indicating which mite species are accidental and which potentially interact with the bird host or benefit from the nesting conditions.

One such benefit may be an increase of mite dispersal potential. During nest building, by collecting and bringing various kinds of material (mosses, lichens, grass, etc.), wood warblers may play an important role in mite dispersal. Mites themselves cannot actively travel long distances because of their small size and lack of wings. With building material, however, they can be transferred over a distance of several dozen meters or so, and in this way wood warblers collecting nest material from various places may increase the possibility of contact between individuals from different, often isolated, mite populations. Dispersal, both active and passive, has important consequences for the structuring of genetic variation within species (Waters et al. 2020). Thus, unintentional movement of mites with nest material may influence genetic variation and population structure of species as well as their ability to spread and increase local ranges.

To conclude, our study has increased faunistic and ecological knowledge of the mite fauna of wood warbler nests. We have shown that wood warbler nests provide a space for survival of numerous mite species. We found several mesostigmatid species that are characteristic of nests, but most of the recorded species are not typical for bird nests and seem to be accidental. We pointed out the potential importance of wood warbler nests for mite dispersal and for the evolution of interspecific interactions.
